# Amide Proton Transfer-Weighted MR Imaging and Signal Variations in a Rat Model of Lipopolysaccharide-Induced Sepsis-Associated Encephalopathy

**DOI:** 10.3390/metabo15070465

**Published:** 2025-07-09

**Authors:** Donghoon Lee, HyunJu Ryu, Yeon Ji Chae, Hind Binjaffar, Chul-Woong Woo, Dong-Cheol Woo, Do-Wan Lee

**Affiliations:** 1 Faculty of Health Sciences, Higher Colleges of Technology, Fujairah P.O. Box 1626, United Arab Emirates; dlee@hct.ac.ae; 2 Faculty of Health Sciences, Higher Colleges of Technology, Dubai P.O. Box 25026, United Arab Emirates; hryu@hct.ac.ae (H.R.); hbinjaffar@hct.ac.ae (H.B.); 3 Department of Convergence Medicine, University of Ulsan College of Medicine, Asan Medical Center, Seoul 05505, Republic of Korea; cyjww1210@gmail.com (Y.J.C.); dcwoo@amc.seoul.kr (D.-C.W.); 4 Convergence Medicine Research Center, Asan Institute for Life Sciences, Asan Medical Center, Seoul 05505, Republic of Korea; wandj79@hanmail.net; 5 Department of Radiology, University of Ulsan College of Medicine, Asan Medical Center, Seoul 05505, Republic of Korea

**Keywords:** lipopolysaccharide-induced sepsis, hippocampus, rat model, amide proton transfer-weighted imaging

## Abstract

**Introduction:** Sepsis-associated encephalopathy (SAE) is an acute brain dysfunction secondary to systemic infection, occurring without direct central nervous system involvement. Despite its clinical relevance, reliable biomarkers for diagnosing SAE and assessing its severity remain limited. This study aimed to evaluate the feasibility of amide proton transfer-weighted (APTw) chemical exchange saturation transfer (CEST) MRI as a non-invasive molecular imaging technique for detecting metabolic alterations related to neuroinflammation in SAE. Using a lipopolysaccharide (LPS)-induced rat model, we focused on hippocampal changes associated with neuronal inflammation. **Materials and Methods:** Twenty-one Sprague–Dawley rats (8 weeks old, male) were divided into three groups: control (CTRL, *n* = 7), LPS-induced sepsis at 5 mg/kg (LPS05, *n* = 7), and 10 mg/kg (LPS10, *n* = 7). Sepsis was induced via a single intraperitoneal injection of LPS. APTw imaging was performed using a 7 T preclinical MRI system, and signal quantification in the hippocampus was conducted using the magnetization transfer ratio asymmetry analysis. **Results and Discussion:** APTw imaging at 7 T demonstrated significantly elevated hippocampal APTw signals in SAE model rats (LPS05 and LPS10) compared to the control (CTRL) group: CTRL (−1.940 ± 0.207%) vs. LPS05 (−0.472 ± 0.485%) (*p* < 0.001) and CTRL vs. LPS10 (−0.491 ± 0.279%) (*p* < 0.001). However, no statistically significant difference was observed between the LPS05 and LPS10 groups (*p* = 0.994). These results suggest that APTw imaging can effectively detect neuroinflammation-related metabolic alterations in the hippocampus. **Conclusion:** Our findings support the feasibility of APTw CEST imaging as a non-invasive molecular MRI technique for SAE, with potential applications in diagnosis, disease monitoring, and therapeutic evaluation.

## 1. Introduction

Sepsis-associated encephalopathy (SAE) refers to a rapid onset of brain dysfunction triggered by systemic infection, occurring in the absence of direct central nervous system (CNS) involvement [1,2]. It manifests as symptoms such as altered consciousness, confusion, cognitive impairment, seizures, or profound coma [3,4,5,6]. Moreover, SAE is associated with higher mortality rates, prolonged hospitalization, and persistent cerebral biomolecular changes [7,8,9]. While its diagnosis relies on clinical evaluation, there remains an absence of reliable biomarkers to definitively detect, confirm, or quantify the severity of SAE [8,10,11]. Consequently, identifying robust biomarkers that reflect the neuro-biological alterations in the brain induced by SAE and hold potential for future diagnostic applications represents a critical area of research.

Molecular imaging has significantly enhanced our ability to study and interpret complex biochemical phenomena [12,13,14]. This noninvasive approach enables the precise visualization of cellular and molecular activities in living cells, tissues, or intact organisms, providing invaluable insights into biological processes [15,16]. Additionally, molecular imaging provides robust tools for quantifying disease-related changes and mapping metabolic alterations within specific target regions, thereby deepening our understanding of underlying pathological mechanisms [17,18]. In our previous studies, we have utilized glutamate-weighted chemical exchange saturation transfer (GluCEST) [19], multi-parametric magnetic resonance imaging (mpMRI) [20] to characterize neurochemical and functional changes in SAE models. In addition to our findings, other molecular imaging techniques such as proton MR spectroscopy (^1^H-MRS) [21,22,23], resting-state functional MRI (fMRI) [21], dynamic positron emission tomography/computed tomography (dPET/CT) [24], and PET [25] have also demonstrated utility in assessing disease severity, monitoring therapeutic responses, and enhancing diagnostic accuracy in SAE.

CEST is a molecular MRI technique that enables the indirect detection of proton exchange between solutes and water, enhancing the visualization of endogenous molecules with protons resonating at distinct frequency offsets from water [26]. Signal attenuation in CEST arises from the saturation of mobile solute protons and is modulated by factors such as the concentration of exchangeable protons, the exchange rate, pH, and other mechanisms, including magnetization transfer (MT) and nuclear Overhauser enhancement (NOE) [27]. In vivo CEST imaging has been extensively applied to detect a variety of compounds, including amide protons, glutamate, and glycosaminoglycans. This technique has proven valuable in investigating numerous brain disorders, such as tumors [28], epileptic seizure [29,30], SAE [19], multiple sclerosis [31,32], and several psychiatric conditions [33,34]. A specialized form of CEST, amide proton transfer-weighted (APTw) MRI, provides high-resolution molecular contrast by capturing amide proton exchange processes [35,36,37]. It is well-recognized for identifying key biomarkers in diseases like tumors and stroke and has recently shown potential in studying neurological inflammatory disorders, with emerging evidence supporting its broader applicability [38,39].

The objective of this study is to quantitatively evaluate changes in APTw signals in an animal model of SAE using well-established in vivo CEST imaging at 7 T. Additionally, this study aims to visualize metabolic alterations in specific target regions of interest (ROIs) within the brain. By employing APTw imaging, we seek to provide detailed insights into the biochemical environment of the brain under SAE conditions, facilitating a deeper understanding of the metabolic disruptions associated with this condition. This approach offers the potential to enhance the diagnostic and therapeutic strategies for managing SAE by highlighting precise metabolic changes within the brain.

## 2. Materials and Methods

### 2.1. Ethics Statement

To minimize the number of animals used and to mitigate any pain and suffering, all experimental procedures were meticulously designed. This study was conducted in accordance with the principles set forth in the Animal Research: Reporting of In Vivo Experiments (ARRIVE) guidelines and the Basel Declaration, which emphasize the 3R concept (Replacement, Reduction, and Refinement). Moreover, all protocols involving animal care and experimental procedures received approval from the Animal Care and Use Committee of the Asan Medical Center, University of Ulsan College of Medicine.

**Figure 1:**
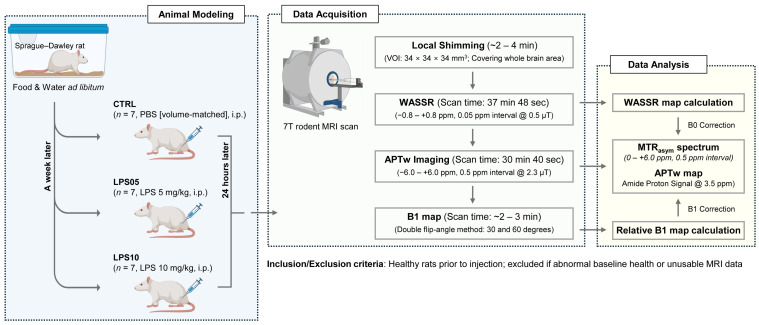
Diagram of the experimental workflow from animal modeling to data analysis. All animals (Sprague–Dawley rats) are acclimatized with food and water ad libitum and then randomly assigned to three groups: CTRL (*n* = 7, PBS volume-matched, i.p.), LPS05 (*n* = 7, LPS 5 mg/kg, i.p.), and LPS10 (*n* = 7, LPS 10 mg/kg, i.p.). Twenty-four hours after injection, 7 T MRI is performed, including WASSR, APTw, and B1 mapping. The resulting data are processed to generate B0- and B1-corrected APTw maps. Abbreviations: APTw, amide proton transfer-weighted; CTRL, control; LPS, lipopolysaccharide; i.p., intraperitoneal; MRI, magnetic resonance imaging; MTR_asym_, magnetization transfer ratio asymmetry; and WASSR, water saturation shift referencing. Created in BioRender. Do-Wan Lee. (2025) https://app.biorender.com/biorender-templates.

An overview of the experimental workflow, including animal modeling, data acquisition, and data analysis, is illustrated in Figure 1.

### 2.2. LPS-Induced Sepsis Modeling

At 8 weeks of age, 21 male Sprague–Dawley rats were obtained from Orient Bio, Inc. (Seongnam, Kyunggi-do, Korea) and assigned into three experimental groups: two lipopolysaccharide (LPS)-induced groups (LPS05, *n* = 7 and LPS10, *n* = 7) and a control group (CTRL, *n* = 7). The rats were pair-housed in standard plastic cages under a 12 h light–dark cycle at an ambient temperature of 23–24 °C. Before initiating the experiments, the rats were acclimatized for one week with free access to food and water. SAE was induced by a single intraperitoneal injection of LPS (lipopolysaccharides from Escherichia coli O26; L8274; Sigma Aldrich, St. Louis, MO, USA) at doses of 5 mg/kg (LPS05) and 10 mg/kg (LPS10) [19,20,40,41]. The control group received an equal volume of sterile endotoxin-free phosphate-buffered saline intraperitoneally. MRI scans were performed on all animals 24 h after LPS or vehicle administration.

### 2.3. Clinically Informed Observations in LPS-Treated Rats

Although formal behavioral or neurological scoring was not performed, general clinical observations were made throughout this study. Rats treated with LPS (5 or 10 mg/kg, i.p.) commonly exhibited reduced locomotor activity, piloerection, hunched posture, and minor hemorrhagic signs around the eyes and nose. These signs typically emerged within hours of LPS injection and persisted up to 24 h. Such manifestations are consistent with systemic inflammation and sepsis-associated encephalopathy (SAE) in rodent models, as reported in previous studies.

### 2.4. In Vivo APTw CEST Imaging

MRI studies were conducted using a 7 T horizontal-bore PharmaScan 70/16 scanner (Bruker BioSpin GmbH, Ettlingen, Germany), featuring a 400 mT/m self-shielding gradient system and an actively decoupled cross-coil setup, which included a 72 mm body coil for signal excitation and a 25 mm single-loop surface coil specifically for imaging the rat brain. Anesthesia was administered via medical air (1.0 L/min) combined with isoflurane (1.5–3% for induction and 2% for maintenance), mixed with 75% N_2_O and 25% O_2_, delivered through a nose cone during the scans. The rats were secured using ear bars, and their body temperature was regulated at approximately 37 °C using a warm-water circulation system incorporated into the scanner bed. Continuous monitoring of physiological parameters, such as heart rate and respiratory rate, was performed using an animal respiratory gating system (SA Instruments, Stony Brook, NY, USA) [42].

Prior to APTw imaging scans, localized high-order shimming was performed using a 34 × 34 × 34 mm^3^ ROI encompassing the entire brain to ensure a uniform magnetic field and optimal signal-to-noise ratio within the MRI system. APTw data acquisition employed a fat-suppressed turbo-rapid acquisition with relaxation enhancement (RARE) sequence with the following parameters: repetition time (TR) of 4.2 s, echo time (TE) of 36.4 milliseconds, RARE factor of 16, matrix size of 96 × 96, field of view (FOV) of 30 × 30 mm^2^, and slice thickness of 1.5 mm. Continuous-wave radiofrequency (RF) saturation was applied with an RF saturation power of 2.3 μT and an RF saturation duration of 5 s [43]. Each rat scan took approximately 30 min and 40 s. Z-spectra were acquired with 25 frequency offsets ranging from −6 to +6 ppm at 0.5 ppm intervals, along with one unsaturated image (S0). To correct for B0 field inhomogeneity, water saturation shift referencing (WASSR) z-spectra were obtained using 33 frequency offsets from −0.8 to +0.8 ppm at 0.05 ppm intervals with an RF saturation power of 0.5 μT [44]. Both APTw and WASSR scans were conducted on a single two-dimensional coronal slice that provided a clear view of the left and right hippocampal regions.

### 2.5. Data Processing

Data quantification and image reconstruction were carried out using MATLAB R2023b (The MathWorks, Natick, MA, USA). To address B0 field inhomogeneity, the Z-spectra obtained from WASSR data for each voxel were fitted using a 12th-order polynomial function and interpolated at a 0.01 ppm offset resolution. The water signal was adjusted to 0 ppm to acquire frequency shift information for each voxel. Subsequently, the APTw Z-spectra normalized to S0 for each voxel were interpolated at a higher resolution of 0.01 ppm and corrected based on the fitted WASSR Z-spectra. The corrected APTw Z-spectra were then resampled back to the 25 original sampling points.

The APTw signal was calculated by subtracting the magnetization transfer ratio (MTR) at −3.5 ppm upfield from the water signal from that at +3.5 ppm downfield. The formula used was as follows: APTw = MTR_asym_(3.5 ppm) = MTR(+3.5 ppm) − MTR(−3.5 ppm), where MTR (magnetization transfer ratio) is defined as MTR = 1 − S_sat_/S_0_, with S_sat_ and S_0_ representing the signal intensities with and without RF saturation, respectively [42,45]. The APTw signals for all experimental groups were quantified based on manually delineated ROIs in the left and right hippocampus.

### 2.6. Statistical Analysis

All statistical computations were carried out with PASW Statistics 18.0 (SPSS Inc., Chicago, IL, USA). The APTw signals were independently quantified for the left and right hippocampal regions. To assess the normality of the APTw data across the three groups, Kolmogorov–Smirnov tests were employed, with all *p* values exceeding 0.05 (*p* = 0.200 for the Kolmogorov–Smirnov test), confirming a normal distribution. Variations in APTw signals among the three groups (CTRL, LPS05, and LPS10) were evaluated using one-way analysis of variance (ANOVA) followed by Tukey’s post hoc tests for multiple comparisons. Additionally, independent *t*-tests were used to compare APTw values between the left and right hippocampal regions within each group, revealing no significant differences in signal intensities between the two sides. All statistical tests were conducted with a significance threshold set at *p* < 0.05.

## 3. Results

**Figure 2:**
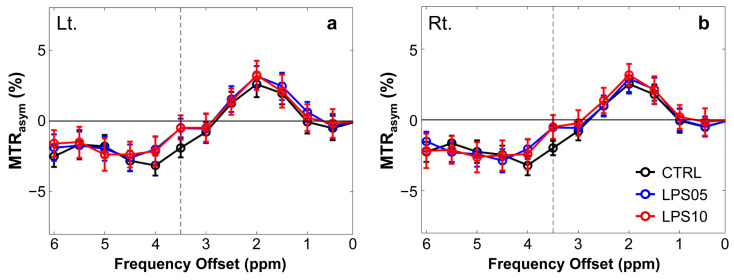
Magnetization transfer ratio asymmetry (MTR_asym_) spectra plotted against frequency offset (ppm) for the left (Lt., **a**) and right (Rt., **b**) hippocampal regions in the control group (CTRL, black, *n* = 7) and the sepsis-associated encephalopathy-induced groups treated with 5 mg/kg (LPS05, blue, *n* = 7) and 10 mg/kg (LPS10, red, *n* = 7) of lipopolysaccharide (LPS). The dotted line at 3.5 ppm indicates the reference point for quantifying amide proton transfer-weighted (APTw) signals. Data points represent mean values ± standard error of the mean (SEM).

Figure 2 shows the MTR_asym_ spectra of APTw signals at 3.5 ppm for the left (Lt., a) and right (Rt., b) hippocampal regions across the three experimental groups: CTRL, LPS05, and LPS10. The MTR_asym_ values were plotted against frequency offsets (ppm) to evaluate differences in APTw signals. In both the left and right hippocampal regions, the LPS05 and LPS10 groups exhibited higher MTR_asym_ values at 3.5 ppm than in the CTRL group.

**Figure 3:**
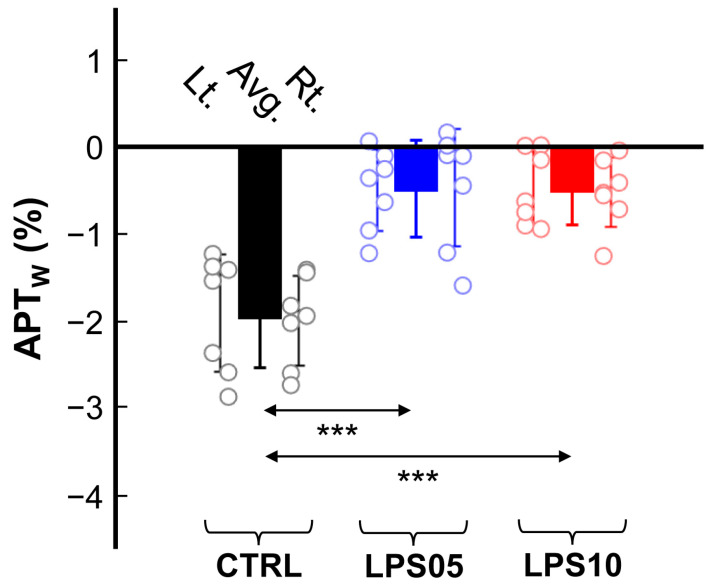
Quantified amide proton transfer-weighted (APTw) signals in the left (Lt.) and right (Rt.) hippocampal regions of the control group (CTRL, black, *n* = 7) and the sepsis-associated encephalopathy-induced groups treated with 5 mg/kg (LPS05, blue, *n* = 7) and 10 mg/kg (LPS10, red, *n* = 7) of lipopolysaccharide (LPS). The solid bars represent the average APTw values for the left and right hippocampus. The error bars denote the standard error of the mean (SEM). Individual data points are displayed as open circles. Significant differences between groups are indicated (*** *p* < 0.001).

Figure 3 presents the quantified APTw signals (%) for the left, right, and averaged (Avg.) hippocampal regions across the three experimental groups. The APTw signals in the LPS05 and LPS10 groups were significantly higher than those in the CTRL group: CTRL (−1.940 ± 0.207%) vs. LPS05 (−0.472 ± 0.485%) (*p* < 0.001) and CTRL vs. LPS10 (−0.491 ± 0.279%) (*p* < 0.001). However, no statistically significant differences were observed between the LPS05 and LPS10 groups (*p* = 0.994). Additionally, independent *t*-tests comparing APTw signals between the left and right hippocampal regions within each group revealed no significant differences: CTRL (Lt. vs. Rt., *p* = 0.795), LPS05 (Lt. vs. Rt., *p* = 0.930), and LPS10 (Lt. vs. Rt., *p* = 0.839).

**Figure 4:**
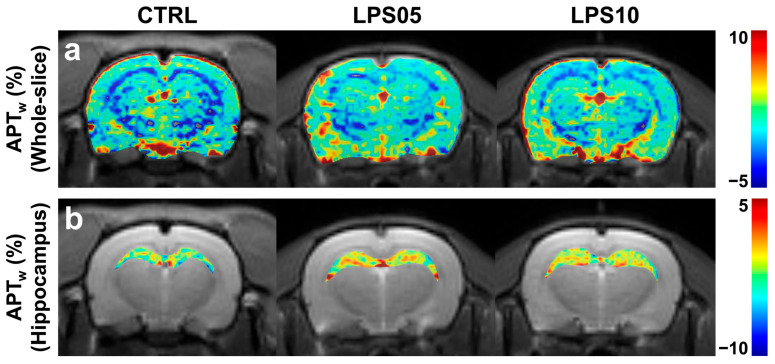
Reconstructed and overlaid amide proton transfer-weighted (APTw) images on unsaturated T2-weighted images of the whole brain (**a**) and hippocampus (**b**) in representative rats from the control group (CTRL, black, *n* = 7) and sepsis-associated encephalopathy-induced groups treated with 5 mg/kg (LPS05, blue, *n* = 7) and 10 mg/kg (LPS10, red, *n* = 7) of lipopolysaccharide (LPS). The color bar indicates the range of calculated APTw signals, with values representing percentage changes.

Figure 4 displays reconstructed maps showing quantified APTw values overlaid on corresponding S0 images for the whole brain (a) and the hippocampal regions (b) across the three experimental groups. In the hippocampal region-specific APTw maps (b), the CTRL group exhibited low and consistent APTw signal intensities across the hippocampus. In contrast, the LPS05 and LPS10 groups demonstrated markedly higher APTw signals in the hippocampal regions.

## 4. Discussion

In this study, we used a well-established LPS-induced rat model of SAE to characterize neuroinflammatory abnormalities in the hippocampus. To capture molecular-level changes in cerebral amide proton signals, we employed in vivo APTw CEST imaging as a molecular MRI technique. APTw signals in the hippocampus were significantly higher in both LPS05 and LPS10 groups compared to the CTRL group; however, no significant difference was observed between the two LPS doses. These findings underscore the utility of APTw imaging in detecting neuroinflammation in SAE. This technique may allow for the quantification of amide proton signal changes, potentially supporting future diagnostic and therapeutic applications.

To the best of our knowledge, this is the first study to apply APTw CEST imaging in an in vivo SAE model. Our results suggest that APTw imaging is sensitive to inflammation-related molecular alterations, potentially reflecting increased amide proton concentrations in affected brain regions. This technique offers a novel window into early neuroinflammatory processes and provides a foundation for further investigation into the biological mechanisms underlying amide proton signal changes in sepsis-induced brain injury.

Previous studies have shown that the APT effect in tissues is primarily governed by the concentration and exchange rate of mobile amide protons [27,46]. However, other physiological factors—such as water proton density and longitudinal relaxation time (T1w)—can also modulate APT signal characteristics [27,46,47]. For instance, Lee et al. reported that both water content and T1w were elevated in tumor regions compared to normal brain tissue [47]. Interestingly, the increased T1w effect was largely compensated by the concurrent rise in water content, suggesting that their combined influence may not significantly distort APTw contrast [27]. This theoretical compensation aligns with our findings, where elevated APTw signals in the LPS-induced groups are likely to reflect true increases in mobile amide proton concentrations, rather than being artifacts of relaxation-related effects [47].

In our previous study using mpMRI, T1 and apparent diffusion coefficient (ADC) values were significantly elevated in the hippocampal regions of LPS-induced SAE models, whereas cerebral blood flow (CBF) and T2 values remained unchanged [20]. The increase in T1 is indicative of altered tissue properties commonly associated with neuroinflammation, such as elevated water content or inflammation-induced edema [47,48,49]. These changes are consistent with SAE-related vascular and interstitial disturbances [50]. Importantly, based on both our current APTw findings and prior mpMRI results [20,45,47], the enhanced APTw signal observed in the hippocampus likely reflects an increase in mobile amide proton concentration due to inflammatory responses [47]. Although T1 prolongation can theoretically enhance APT contrast, the prior literature suggests that its influence may be counterbalanced by concomitant increases in water content, thereby minimizing direct confounding effects on APTw quantification [47]. This supports the interpretation that the observed APTw elevation more specifically reflects molecular-level changes related to protein metabolism in SAE.

Notably, our previous study using the same SAE model demonstrated significantly elevated T1 values in the hippocampus under identical experimental conditions [20], consistent with inflammation-induced changes such as increased water content or tissue edema. Although we discussed compensatory effects between T1 prolongation and water content increase, the absence of direct T1 or water measurements in the current study limits our ability to fully exclude their potential influence on APTw contrast. Future studies should therefore incorporate quantitative T1 mapping and water content analysis to further clarify the specificity of APTw signal alterations in the context of neuroinflammation.

While our findings provide preliminary insights into conditions involving neuronal inflammation and infection, several limitations must be acknowledged. First, although two different LPS doses (5 and 10 mg/kg) were selected based on their known ability to induce systemic inflammation without excessive mortality, the absence of a clear dose–response effect in APTw signals suggests a possible ceiling effect. This may be due not only to the limited dose range but also to statistical underpowering, raising the possibility of a type II error caused by the modest sample size. Additionally, differences in molecular sensitivity across imaging modalities may contribute to this observation. For instance, our previous study using GluCEST imaging demonstrated significant hippocampal glutamate-weighted signal differences between these same LPS doses, indicating that the selected dose range is sufficient to detect neuroinflammatory changes via GluCEST MRI [19]. In contrast, APTw imaging may require finer dose increments (e.g., 7.5 or 12.5 mg/kg) or different imaging timepoints to capture more nuanced signal variations, possibly reflecting its distinct sensitivity to protein-related metabolic changes. Future studies should therefore consider increasing the number of animals per group and expanding the dose range to improve statistical robustness and clarify potential dose–response relationships in APTw imaging.

Second, the limited spatial coverage of APTw CEST imaging may have excluded other brain regions relevant to SAE pathology. While we focused on a single coronal slice of the hippocampus, this region was selected based on substantial prior evidence indicating its heightened vulnerability to neuroinflammatory insults, including in sepsis-associated encephalopathy. The hippocampus is particularly susceptible due to its high metabolic demand, dense glutamatergic transmission, and sensitivity to oxidative and inflammatory stress. Previous preclinical and clinical studies have consistently reported hippocampal damage, microglial activation, and altered neurochemical metabolism in SAE models [51,52,53]. Future studies should employ multi-slice or whole-brain APTw protocols to expand the assessment beyond the hippocampus and capture signal variations in other SAE-relevant regions such as the cortex and thalamus.

Third, this study lacked hematological and histopathological validation to confirm SAE severity or to correlate imaging findings with underlying pathology. This limitation may render some of the biological interpretations of APTw signal changes speculative. Integrating molecular and cellular analyses in future studies will be essential to strengthen the biological validity of APTw-based imaging biomarkers. For example, the inclusion of immunohistochemical markers of neuroinflammation, such as Iba-1 (for microglial activation) and GFAP (for astrocyte reactivity), alongside serum cytokine profiling and systemic inflammatory indices, could help elucidate the underlying mechanisms of APTw signal alterations. Addressing these limitations will enhance the translational relevance of APTw imaging in sepsis-associated encephalopathy and related neuroinflammatory conditions.

Fourth, ROI-based analysis was conducted manually in this study. Although all ROIs were drawn by a single operator blinded to group allocation and consistently placed using anatomical landmarks and predefined dimensions, manual selection remains inherently susceptible to operator-dependent variability. To reduce subjectivity and enhance reproducibility, future studies should consider incorporating semi-automated or fully automated image segmentation tools for ROI definition. These methods could improve the objectivity of signal quantification and are particularly important for translating APTw imaging techniques to clinical settings.

In addition to its feasibility in detecting SAE-associated neuroinflammation, APTw imaging presents complementary value when used alongside other molecular imaging modalities. GluCEST imaging enables the visualization of glutamate concentration changes, while mpMRI offers insights into structural, diffusion, and perfusion abnormalities. In contrast, APTw imaging uniquely reflects endogenous protein and amide proton signal changes, providing a distinct metabolic perspective that is not accessible through GluCEST or mpMRI alone. The integration of APTw with GluCEST and mpMRI could enhance the sensitivity and specificity of neuroinflammatory assessments in both preclinical and clinical settings. As such, APTw holds promise as a valuable component in multi-parametric imaging strategies aimed at improving diagnosis, monitoring, and treatment evaluation in SAE.

## 5. Conclusions

In conclusion, this study presents the first in vivo investigation of SAE using APTw CEST imaging, demonstrating significantly elevated APTw signals in the hippocampal regions of LPS-induced SAE models compared to controls. These findings support the potential of APTw imaging as a non-invasive molecular MRI technique for visualizing metabolic changes associated with neuroinflammation. Although no significant differences were observed between the LPS05 and LPS10 groups, this highlights the importance of optimizing dosing strategies and improving sensitivity to detect dose-dependent effects. The results provide a foundational step toward the application of APTw imaging in preclinical neuroinflammation research. With further validation and integration with other imaging biomarkers or histopathological data, APTw imaging may also hold promise for future clinical use in diagnosing, monitoring, and evaluating treatment responses in patients with sepsis-associated encephalopathy and related neuroinflammatory conditions.

Furthermore, given its sensitivity to endogenous protein alterations, APTw imaging could also be applied to other neurological diseases characterized by neuroinflammation or metabolic dysregulation, such as multiple sclerosis, Alzheimer’s disease, or traumatic brain injury. Future studies exploring these applications may help expand the utility of APTw as a versatile molecular MRI technique across a broad spectrum of neurological disorders.

## Data Availability

The data that support the findings of this study are available from the corresponding author upon reasonable request.

## References

[1] Catarina A.V., Branchini G., Bettoni L., De Oliveira J.R., Nunes F.B. (2021). Sepsis-associated encephalopathy: From pathophysiology to progress in experimental studies. Mol. Neurobiol..

[2] Yang Y., Liang S., Geng J., Wang Q., Wang P., Cao Y., Li R., Gao G., Li L. (2020). Development of a nomogram to predict 30-day mortality of patients with sepsis-associated encephalopathy: A retrospective cohort study. J. Intensive Care.

[3] Chen J., Shi X., Diao M., Jin G., Zhu Y., Hu W., Xi S. (2020). A retrospective study of sepsis-associated encephalopathy: Epidemiology, clinical features and adverse outcomes. BMC Emerg. Med..

[4] Barichello T., Generoso J.S., Collodel A., Petronilho F., Dal-Pizzol F. (2021). The blood-brain barrier dysfunction in sepsis. Tissue Barriers.

[5] Danielski L.G., Giustina A.D., Badawy M., Barichello T., Quevedo J., Dal-Pizzol F., Petronilho F. (2018). Brain Barrier Breakdown as a Cause and Consequence of Neuroinflammation in Sepsis. Mol. Neurobiol..

[6] Buras J.A., Holzmann B., Sitkovsky M. (2005). Animal models of sepsis: Setting the stage. Nat. Rev. Drug Discov..

[7] Bauer M., Gerlach H., Vogelmann T., Preissing F., Stiefel J., Adam D. (2020). Mortality in sepsis and septic shock in Europe, North America and Australia between 2009 and 2019—Results from a systematic review and meta-analysis. Crit. Care.

[8] Bircak-Kuchtova B., Chung H.-Y., Wickel J., Ehler J., Geis C. (2023). Neurofilament light chains to assess sepsis-associated encephalopathy: Are we on the track toward clinical implementation?. Crit. Care.

[9] Cecconi M., Evans L., Levy M., Rhodes A. (2018). Sepsis and septic shock. Lancet.

[10] Sonneville R., Verdonk F., Rauturier C., Klein I.F., Wolff M., Annane D., Chretien F., Sharshar T. (2013). Understanding brain dysfunction in sepsis. Ann. Intensive Care.

[11] Netea M.G., Van Der Meer J.W., Van Deuren M., Kullberg B.J. (2003). Proinflammatory cytokines and sepsis syndrome: Not enough, or too much of a good thing?. Trends Immunol..

[12] James M.L., Gambhir S.S. (2012). A molecular imaging primer: Modalities, imaging agents, and applications. Physiol. Rev..

[13] Stubbs D.J., Yamamoto A.K., Menon D.K. (2013). Imaging in sepsis-associated encephalopathy—Insights and opportunities. Nat. Rev. Neurol..

[14] Kitagawa Y., Nakaso K., Horikoshi Y., Morimoto M., Omotani T., Otsuki A., Inagaki Y., Sato H., Matsura T. (2019). System x_c_^−^ in microglia is a novel therapeutic target for post-septic neurological and psychiatric illness. Sci. Rep..

[15] Osborn E.A., Jaffer F.A. (2008). Advances in molecular imaging of atherosclerotic vascular disease. Curr. Opin. Cardiol..

[16] Weissleder R., Pittet M.J. (2008). Imaging in the era of molecular oncology. Nature.

[17] Dumbuya J.S., Li S., Liang L., Zeng Q. (2023). Paediatric sepsis-associated encephalopathy (SAE): A comprehensive review. Mol. Med..

[18] Mu C., Reed J.L., Wang F., Yan X., Lu M., Gore J.C., Chen L.M. (2025). Validation of qMT and CEST MRI as Biomarkers of Response to Treatment After Lumbar Spinal Cord Injury in Rats. NMR Biomed..

[19] Lee D.-W., Kwon J.-I., Heo H., Woo C.-W., Yu N.H., Kim K.W., Woo D.-C. (2023). Cerebral Glutamate Alterations Using Chemical Exchange Saturation Transfer Imaging in a Rat Model of Lipopolysaccharide-Induced Sepsis. Metabolites.

[20] Lee D., Heo H., Woo C.-W., Chae Y.J., Choi M.Y., Min J., Woo D.-C., Lee D.-W. (2025). In Vivo Assessment of Cerebral Functional Changes in a Rat Model of Sepsis-Associated Encephalopathy Using Multi-Parametric MR Imaging. Appl. Magn. Reson..

[21] Li H., Liao H., Zhang C., Xu Y., Xu X., Chen Y., Song S., Li Q., Si Y., Bao H. (2022). Disrupted metabolic and spontaneous neuronal activity of hippocampus in sepsis associated encephalopathy rats: A study combining magnetic resonance spectroscopy and resting-state functional magnetic resonance imaging. Front. Neurosci..

[22] Liu S., Liu Z., Wu G., Ye H., Wu Z., Yang Z., Jiang S. (2024). Assessment of sepsis-associated encephalopathy by quantitative magnetic resonance spectroscopy in a rat model of cecal ligation and puncture. Heliyon.

[23] Wen M., Lian Z., Huang L., Zhu S., Hu B., Han Y., Deng Y., Zeng H. (2017). Magnetic resonance spectroscopy for assessment of brain injury in the rat model of sepsis. Exp. Ther. Med..

[24] Zhu T., Jiang J., Xiao Y., Xu D., Liang Z., Bi L., Yang M., Liang M., Li D., Lin Y. (2022). Early Diagnosis of Murine Sepsis-Associated Encephalopathy Using Dynamic PET/CT Imaging and Multiparametric MRI. Mol. Imaging Biol..

[25] Szöllősi D., Hegedűs N., Veres D.S., Futó I., Horváth I., Kovács N., Martinecz B., Dénes Á., Seifert D., Bergmann R. (2018). Evaluation of Brain Nuclear Medicine Imaging Tracers in a Murine Model of Sepsis-Associated Encephalopathy. Mol. Imaging Biol..

[26] Van Zijl P.C., Yadav N.N. (2011). Chemical exchange saturation transfer (CEST): What is in a name and what isn’t?. Magn. Reson. Med..

[27] Zhou J., Payen J.-F., Wilson D.A., Traystman R.J., Van Zijl P.C.M. (2003). Using the amide proton signals of intracellular proteins and peptides to detect pH effects in MRI. Nat. Med..

[28] Zhou J., Tryggestad E., Wen Z., Lal B., Zhou T., Grossman R., Wang S., Yan K., Fu D.-X., Ford E. (2011). Differentiation between glioma and radiation necrosis using molecular magnetic resonance imaging of endogenous proteins and peptides. Nat. Med..

[29] Lee D.-H., Lee D.-W., Kwon J.-I., Kim S.-T., Woo C.-W., Kim J.K., Kim K.W., Lee J.S., Choi C.G., Suh J.-Y. (2019). Changes to gamma-aminobutyric acid levels during short-term epileptiform activity in a kainic acid-induced rat model of status epilepticus: A chemical exchange saturation transfer imaging study. Brain Res..

[30] Lee D.-H., Lee D.-W., Kwon J.-I., Woo C.-W., Kim S.-T., Lee J.S., Choi C.G., Kim K.W., Kim J.K., Woo D.-C. (2019). In Vivo Mapping and Quantification of Creatine Using Chemical Exchange Saturation Transfer Imaging in Rat Models of Epileptic Seizure. Mol. Imaging Biol..

[31] Lee D.-W., Woo D.-C., Heo H., Kim K.W., Kim J.K., Lee D.-H. (2020). Signal alterations of glutamate-weighted chemical exchange saturation transfer MRI in lysophosphatidylcholine-induced demyelination in the rat brain. Brain Res. Bull..

[32] Lee D.W., Heo H., Woo C.W., Woo D.C., Kim J.K., Kim K.W., Lee D.H. (2020). Temporal changes in in vivo glutamate signal during demyelination and remyelination in the corpus callosum: A glutamate-weighted chemical exchange saturation transfer imaging study. Int. J. Mol. Sci..

[33] Lee D., Woo C.-W., Heo H., Ko Y., Jang J.S., Na S., Kim N., Woo D.-C., Kim K.W., Lee D.-W. (2024). Mapping Changes in Glutamate with Glutamate-Weighted MRI in Forced Swim Test Model of Depression in Rats. Biomedicines.

[34] Qi K., Li H., Tao J., Liu M., Zhang W., Liu Y., Liu Y., Gong H., Wei J., Wang A. (2025). Glutamate chemical exchange saturation transfer (GluCEST) MRI to evaluate the relationship between demyelination and glutamate content in depressed mice. Behav. Brain Res..

[35] Foo L.S., Harston G., Mehndiratta A., Yap W.-S., Hum Y.C., Lai K.W., Mukari S.A.M., Zaki F.M., Tee Y.K. (2021). Clinical translation of amide proton transfer (APT) MRI for ischemic stroke: A systematic review (2003–2020). Quant. Imaging Med. Surg..

[36] Cronin A.E., Liebig P., Detombe S.A., Duggal N., Bartha R. (2024). Reproducibility of 3D chemical exchange saturation transfer (CEST) contrasts in the healthy brain at 3T. Sci. Rep..

[37] Hamon G., Lecler A., Ferré J.-C., Bourdillon P., Duron L., Savatovsky J. (2024). 3-Tesla amide proton transfer-weighted imaging (APT-WI): Elevated signal also in tumor mimics. Eur. Radiol..

[38] By S., Barry R.L., Smith A.K., Lyttle B.D., Box B.A., Bagnato F.R., Pawate S., Smith S.A. (2018). Amide proton transfer CEST of the cervical spinal cord in multiple sclerosis patients at 3T. Magn. Reson. Med..

[39] Liu Z., Yang Q., Liu H., Luo H., Zheng Y., Luo D., Wu Y. (2025). Mitigation of T1 impact for unbiased tumor magnetic resonance amide proton transfer imaging at 3T. Radiol. Adv..

[40] Liu P.-P., Yu X.-Y., Pan Q.-Q., Ren J.-J., Han Y.-X., Zhang K., Wang Y., Huang Y., Ban T. (2025). Multi-Omics and Network-Based Drug Repurposing for Septic Cardiomyopathy. Pharmaceuticals.

[41] Peek V., Harden L.M., Damm J., Aslani F., Leisengang S., Roth J., Gerstberger R., Meurer M., von Köckritz-Blickwede M., Schulz S. (2021). LPS primes brain responsiveness to high mobility group box-1 protein. Pharmaceuticals.

[42] Lee D.-W., Heo H., Woo D.-C., Kim J.K., Lee D.-H. (2021). Amide proton transfer-weighted 7-T MRI contrast of myelination after cuprizone administration. Radiology.

[43] Sagiyama K., Mashimo T., Togao O., Vemireddy V., Hatanpaa K.J., Maher E.A., Mickey B.E., Pan E., Sherry A.D., Bachoo R.M. (2014). In vivo chemical exchange saturation transfer imaging allows early detection of a therapeutic response in glioblastoma. Proc. Natl. Acad. Sci. USA.

[44] Kim M., Gillen J., Landman B.A., Zhou J., Van Zijl P.C. (2009). Water saturation shift referencing (WASSR) for chemical exchange saturation transfer (CEST) experiments. Magn. Reson. Med..

[45] Zhou J., Heo H.Y., Knutsson L., van Zijl P.C., Jiang S. (2019). APT-weighted MRI: Techniques, current neuro applications, and challenging issues. J. Magn. Reson. Imaging.

[46] Zhou J., Lal B., Wilson D.A., Laterra J., Van Zijl P.C. (2003). Amide proton transfer (APT) contrast for imaging of brain tumors. Magn. Reson. Med. Off. J. Int. Soc. Magn. Reson. Med..

[47] Lee D.H., Heo H.Y., Zhang K., Zhang Y., Jiang S., Zhao X., Zhou J. (2017). Quantitative assessment of the effects of water proton concentration and water T1 changes on amide proton transfer (APT) and nuclear overhauser enhancement (NOE) MRI: The origin of the APT imaging signal in brain tumor. Magn. Reson. Med..

[48] Furtmann J.K., Sichtermann T., Oros-Peusquens A.-M., Dekeyzer S., Shah N.J., Wiesmann M., Nikoubashman O. (2022). MRI Analysis Of the Water Content Change In the Brain During Acute Ethanol Consumption Via Quantitative Water Mapping. Alcohol Alcohol..

[49] Nishioku T., Dohgu S., Takata F., Eto T., Ishikawa N., Kodama K.B., Nakagawa S., Yamauchi A., Kataoka Y. (2009). Detachment of brain pericytes from the basal lamina is involved in disruption of the blood–brain barrier caused by lipopolysaccharide-induced sepsis in mice. Cell. Mol. Neurobiol..

[50] Weimer J., Jones S., Frontera J. (2017). Acute cytotoxic and vasogenic edema after subarachnoid hemorrhage: A quantitative MRI study. Am. J. Neuroradiol..

[51] Gofton T.E., Young G.B. (2012). Sepsis-associated encephalopathy. Nat. Rev. Neurol..

[52] Heming N., Mazeraud A., Verdonk F., Bozza F.A., Chrétien F., Sharshar T. (2017). Neuroanatomy of sepsis-associated encephalopathy. Crit. Care.

[53] Ziaja M. (2012). Sepsis and septic encephalopathy: Characteristics and experimental models. Folia Neuropathol..

